# Supplementary material for: Predation and fragmentation portrayed in the statistical structure of prey time series
Source: BMC Ecol. 2009 May 6;9:10. doi: 10.1186/1472-6785-9-10 (PMC2689204; doi:10.1186/1472-6785-9-10)
Supplement: Additional file 2 — Voles and related classes ODDox Documentation. ODDox documentation of the agent-based model (ALMaSS) applied by Hendrichsen et al. The documentation is started by activating main.html. [file 1472-6785-9-10-S2.zip › Vole_ODDox/page0.html]

ALMaSS ODDox: References

- Main Page
- Related Pages
- Classes
- Files

# References

  
  

Agrell, J., Erlinge, S., Nelson, J., Sandell, M., 1996. Shifting spacing behaviour of male field voles (Microtus agrestis) over the reproductive season. Ann. Zool. Fennici 33: 243-248.  |

Agrell, J. Wolff, J.O., Ylonen, H., 1998. Counter-strategies to infanticide in mammals: costs and consequences. Oikos 83: 507-517.  |

Andera, M., 1981, Reproduction of Microtus agrestis in Czechoslovakia. Acta Sci. Nat. Brno 15(5): 1-38 (from "Handbuch der Saugetiere Europas").  |

Erlinge, S., Goransson, G., Hansson, L., Hogstedt, G., Liberg, O., Nilsson, I.N., Nilsson, T., Schantz, T., Sylven, M., 1983. Predation as a regulating factor on small rodent populations in southern Sweden. Oikos 40: 36-52.  |

Erlinge, S., Hoogenboom, I., Agrell, J., Nelson, J., Sandell, M., 1990. Density-related home-range size and overlap in adult field voles (Microtus agrestis) in southern Sweden. J. Mamm. 71, 597-603.  |

Hansson, L., 1971. Habitat, food and population dynamics of the field vole Microtus agrestis (L.) in South Sweden. Viltrevy, Swedish Wildlife, Svenska Jagerforbundet 373p.  |

Hansson, L., 1977. Spatial dynamics of field voles Microtus agrestis in heterogeneous landscapes. Oikos 29: 593-644.  |

Heise, S., Lippke, J., 1997. Role of female aggression in prevention of infanticidal behaviour in male Common Voles, Microtus arvalis (Pallas, 1779). Aggressive Behaviour. 23: 293-298.  |

Grimm, V., Berger, U., Bastiansen, F., Eliassen, S., Ginot, V., Giske, J., Goss-Custard, J., Grand, T., Heinz, S.K., Huse, G., Huth, A., Jepsen, J.U., Jorgensen, C., Mooij, W.M., Muller, B., Pe'er, G., Piou, C., Railsback, S.F., Robbins, A.M., Robbins, M.M., Rossmanith, E., Ruger, N., Strand, E., Souissi, S., Stillman, R.A., Vabo, R., Visser, U., & DeAngelis, D.L. (2006) A standard protocol for describing individual-based and agent-based models. Ecological Modelling, 198, 115-126.

Innes, D.G., Millar, J. S. 1994. Life histories of Clethrionomys and Microtus (Microtinae). Mammal Review. 24 (4): 179-207.  |

Hacklander, K., W. Arnold, & Ruf, T. (2002). "Postnatal development and thermoregulation in the precocial European hare (Lepus europaeus)." Journal of Comparative Physiology B-Biochemical Systemic and Environmental Physiology 172(2): 183-190.

Jensen, T.S., Hansen, T.S. 2001, Effekten af husdyrgaesning paa smaapattedyr. In: Eds: Pedersen, L. B., Buttenschoen, R. M., Petersen, H. and Jensen, T. S. "Graening paa ekstensivt drevne naturarealer - Effekter paa stofkredsloeb og naturindhold". Park- og Landskabsserien nr. 34, Skov & Landskab, Hoersholm: 107-121.  |

Leslie, P.H., Ranson, R.M., 1940. The mortality, fertility and rate of natural increase of the vole (Microtus agrestis) as observed in the laboratory. J. Anim. Ecol. 9: 27-52.  |

Myllymaki, A., 1977. Demographic mechanisms in the fluctuating populations of the field vole Microtus agrestis. Oikos 29: 468-493.  |

Odderskaer P, Topping C J, Petersen M B, Rasmussen J, Dalgaard T, Erlandsen M (2006) Ukrudtsstriglingens effekter paa dyr, planter og ressourceforbrug. Miljeostyrelsen, Bekaempelsesmiddelforskning fra Miljeostyrelsen 105. Available at http://www2.mst.dk/Udgiv/publikationer/2006/87-7052-343-6/pdf/87-7052-344-4.pdf  |

Pielowski, Z. (1971) "Length of life of the hare." Acta Theriologica 16(1-7): 89-94.

Topping, C.J., Rehder, M.J. & Mayoh, B.H. 1999. VIOLA: a new visual programming language designed for the rapid development of interacting agent systems. Acta Biotheoretica. 47: 129?140.  |

Topping C.J., Hansen, T.S., Jensen, T.S., Jepsen, J.U., Nikolajsen, F. and Oddersaer, P. 2003. ALMaSS, an agent-based model for animals in temperate European landscapes. Ecological Modelling 167: 65-82.  |

Topping C.J. & Odderskaer, P. 2004. Modeling the influence of temporal and spatial factors on the assessment of impacts of pesticides on skylarks. Environmental Toxicology & Chemistry 23, 509-520.  |

Topping, C.J., Sibly, R.M., Akcakaya, H.R., Smith, G.C. & Crocker, D.R. 2005: Risk assessment of UK skylark populations using life-history and individual-based landscape models. - Ecotoxicology 14(8): 863-876.  |

---

Generated on Thu Jan 22 14:13:45 2009 for ALMaSS ODDox by 
 1.5.6
